# Mapping the Drivers of Climate Change Vulnerability for Australia’s Threatened Species

**DOI:** 10.1371/journal.pone.0124766

**Published:** 2015-05-27

**Authors:** Jasmine R. Lee, Ramona Maggini, Martin F. J. Taylor, Richard A. Fuller

**Affiliations:** 1 School of Biological Sciences, The University of Queensland, Brisbane, Queensland, Australia; 2 Australian Research Council Centre of Excellence for Environmental Decisions (CEED), The University of Queensland, Brisbane, Queensland, Australia; 3 WWF-Australia, Brisbane, Queensland, Australia; State Natural History Museum, GERMANY

## Abstract

Effective conservation management for climate adaptation rests on understanding the factors driving species’ vulnerability in a spatially explicit manner so as to direct on-ground action. However, there have been only few attempts to map the spatial distribution of the factors driving vulnerability to climate change. Here we conduct a species-level assessment of climate change vulnerability for a sample of Australia’s threatened species and map the distribution of species affected by each factor driving climate change vulnerability across the continent. Almost half of the threatened species assessed were considered vulnerable to the impacts of climate change: amphibians being the most vulnerable group, followed by plants, reptiles, mammals and birds. Species with more restricted distributions were more likely to show high climate change vulnerability than widespread species. The main factors driving climate change vulnerability were low genetic variation, dependence on a particular disturbance regime and reliance on a particular moisture regime or habitat. The geographic distribution of the species impacted by each driver varies markedly across the continent, for example species impacted by low genetic variation are prevalent across the human-dominated south-east of the country, while reliance on particular moisture regimes is prevalent across northern Australia. Our results show that actions to address climate adaptation will need to be spatially appropriate, and that in some regions a complex suite of factors driving climate change vulnerability will need to be addressed. Taxonomic and geographic variation in the factors driving climate change vulnerability highlights an urgent need for a spatial prioritisation of climate adaptation actions for threatened species.

## Introduction

Climate change poses a serious and accelerating threat to species and ecosystems worldwide [1–3]. Along with habitat loss through human land use, climate change is a major contributor to biodiversity loss in the 21^st^ century [4]. Assessments of the extent to which species are vulnerable to climate change allow us to evaluate the relative importance of the threat of climate change against the range of other threats facing species [5–6]. However, while many such assessments exist [7–13], studies tend to focus on a single region, species or taxonomic group and to our knowledge none has yet mapped the individual drivers spatially. As a consequence there remains considerable uncertainty about where and how we should take on-ground action to help vulnerable species adapt to climate change [14].

Several approaches have been used to assess vulnerability to climate change. These range from assessments of climate change processes coupled with literature-based evaluations of how these might affect species or ecosystems [15–16], to the use of species distribution models predicting the change in geographic distribution required for a species to track suitable climatic conditions [8, 17–20]. Another method is to generate climate change vulnerability indices that summarise detailed information on the sensitivity of species to climate change and their adaptive capacity to respond to changing conditions, as well as their exposure to a changing climate [8, 12, 21]. Sensitivity is determined by the adaptive capacity and resilience of a species, and depends on intrinsic traits such as physiological tolerances, biological traits and genetic diversity [21–22]. Exposure expresses the magnitude of the change in the climatic conditions (e.g. temperature, precipitation) within the geographic area occupied by the species. Vulnerability indices are often expressed as a overall measure of the potential harm of climate change to a species or ecosystem and can be summarised in a single number, but because they are built on detailed information about the factors driving climate change vulnerability, they can also be decomposed to reveal spatial and taxonomic variation in the underlying causes of climate change vulnerability. Pinpointing these causes can help begin the process of designing management actions aimed at addressing them. Here we assess which factors drive climate change vulnerability, and how those drivers are distributed spatially. Designing a set of management actions for climate adaptation therefore depends on (i) a clear understanding of the extent to which species are vulnerable to climate change, (ii) knowledge about which aspects of a species’ ecology drive its climate change vulnerability, and (iii) information on how species affected by the various drivers of climate change vulnerability are spatially distributed.

Assessments of species’ vulnerability to climate change have multiplied rapidly following the development of vulnerability assessment toolkits and frameworks (e.g. [23–26]). Gardali et al. [11] used exposure and sensitivity to assess 358 Californian birds, classifying 36% of them as vulnerable to climate change. A recent global assessment based on species traits concluded that 24% of birds, 22% of amphibians and 15% of corals are highly climate change vulnerable under an optimistic climate change scenario, rising to 50%, 44% and 32% respectively under a pessimistic climate change scenario [23]. The same study demonstrated relatively low spatial congruence between the distributions of species with high exposure, high sensitivity and low adaptive capacity, suggesting that different aspects of climate change vulnerability may be important in different places. Here we further expand on this work by mapping the distributions of species affected by individual climate change vulnerability factors.

In this paper we determine the factors driving climate change vulnerability for a representative set of 213 threatened species across Australia. We (i) assess the climate change vulnerability of the species accounting for exposure, sensitivity and adaptive capacity, (ii) identify which species and taxonomic groups are most vulnerable to climate change, and (iii) determine the spatial distribution of species affected by each climate change vulnerability factor. We associate specific climate change vulnerability factors with the areas in which the species occur, indicating which climate adaptation actions (management to conserve species in a changing climate) will be needed in each bioregion across the continent. In so doing, we pave the way for building a spatially explicit prioritisation of management actions to protect threatened species under climate change.

## Methods

### Species assessed

We assessed vulnerability to climate change for a sample of species listed as threatened in Australia’s Environment Protection and Biodiversity Conservation Act (EPCB Act; [27]). All birds (n = 44), mammals (n = 43), amphibians (n = 19) and reptiles (n = 14) with known population trends [28] were selected from this list. We then randomly chose a species from each plant family to form a subset (n = 112) of plant species from the 705 listed plants with known population trends. Maps of the current distribution of the species were obtained from DSEWPaC [29]. We considered only polygons that were identified as having known or likely species occurrences and removed from the analysis polygons where species “may occur”.

### General approach

To estimate each species’ vulnerability to climate change we used the NatureServe climate change vulnerability index [26]. This index was developed according to the framework produced by Williams et al. [21], and integrates information on exposure (six factors: two direct and four indirect, Table 1; for full details on how we converted raw data into categorical scores for indirect exposure factors, see S2 Table) and intrinsic sensitivity to climate change (sixteen factors; Table 1). Based on analysis of relevant literature, we scored each factor according to its contribution to each species’ vulnerability: ‘decrease vulnerability’ (DV), ‘somewhat decrease vulnerability’ (SDV), neutral (N), ‘somewhat increase vulnerability’ (SIV), ‘increase vulnerability’ (IV), ‘greatly increase vulnerability’ (GIV; S1 Dataset). Where there was uncertainty about a classification, we assigned a species to multiple categories as advised by Young et al. [26].

**Table 1:** Factors used to calculate the climate change vulnerability index.

*Category*	*Factor*	*Description*
Direct exposure	Difference in mean annual temperature	Calculated from the proportion of each species’ geographic range affected by each of five different magnitudes of mean annual temperature change across Australia (S1 Table).
	Difference in mean annual moisture index	Calculated from the proportion of each species’ geographic range affected by each of six different magnitudes of annual moisture index change across Australia (S1 Table).
Indirect exposure	Exposure to sea level rise	Exposure of species’ geographic range to areas likely to be inundated by sea level rise.
	Distribution relative to natural barriers	Overlap of a 50km buffer from the edge of the species’ current distributions with natural barriers, comprising highlands, major water bodies and areas devoid of any vegetation.
	Distribution relative to anthropogenic barriers	Overlap of a 50km buffer from the edge of the species’ current distributions with anthropogenic barriers, comprising urban, cultivated and managed areas.
Sensitivity	Dispersal ability	Scored based on the known or predicted dispersal or movement capacity. Species better able to disperse or move long distances are expected to be better able to track suitable climate conditions.
	Reliance on cool temperatures	Scored based on reliance on a cool temperature environment (such as frost pockets, alpine areas or south-facing slopes).
	Reliance on a particular moisture regime or habitat	Scored based on reliance on a seasonal hydrological regime and/or a specific aquatic or wetland habitat or localised moisture regime. For example, some species require a certain amount of rainfall each season, or a certain proximity to standing water.
	Dependence on a specific disturbance regime likely to be impacted by climate change	Scored based on sensitivity to changes in particular disturbance regimes, such as fire or flood, which are likely to change with climate. For example some species rely on fire for reproduction and some on flood for dispersal. Species have increased vulnerability if the altered regime is likely to negatively impact the species (eg. increased frequency of fire).
	Dependence on snow-cover habitats	Scored based on reliance dependance on habitats associated with ice or snow during all or parts of their life cycle (eg. winter hibernation).
	Reliance on a particular abiotic feature or derivatives	Scored based on reliance on, or restriction to, specific abiotic features, particulary where uncommon in the landscape (eg. restriction to sand dunes, caves or a particular soil type).
	Reliance on other species for habitat	Scored based on dependence on other species to provide habitat (eg. relying on particular plant species for breeding or feeding).
	Dietary versatility (animals only); or	Scored based on reliance on a particular taxon for diet (eg. only eats termites).
	Pollinator versatility (plants only)	Scored based on reliance on a particular taxon for pollination.
	Dependence on other species for propagule dispersal	Scored based on reliance on another species to disperse propagules (most animals do not rely on other species in this way).
	Reliance on another species for other interspecfic interaction	Scored based on reliance on another species for a interspecific interaction not covered by habitat, diet, pollinator or propagule dispersal (eg. reliance on a mycorrhizal symbiosis).
	Measured genetic variation (when available); or	Scored based a direct measure of genetic variation. Species have increased vulnerability when their genetic variation has been determined to be low in comparison with related species.
	Occurrence of bottlenecks in recent evolutionary history (measured genetic variation not available)	Scored based on signs of a recent genetic bottleneck, for example severe range contraction or steep population decline.

Note that a species may only be scored on dietary versatility OR pollinator versatility and measured genetic variation OR occurrence of recent population bottlenecks (described in text as ‘low genetic variation’). Owing to limited data availability, in our assessment we did not include ‘predicted impact of land use change resulting from human responses to climate change’,‘historical thermal or hydrological niche’or ‘phenological response to climate change’, which are available for scoring in the original NatureServe index [26].

The indirect exposure and sensitivity factors were combined and weighted by direct exposure to generate a continuous climate change vulnerability index value for each species (for full detail see: [26, 30]). For this purpose scores of each factor (those comprising indirect exposure and sensitivity) were translated into a numerical value (DV = -2, SDV = -1, N = 0, SIV = 1, IV = 2, GIV = 3), where multiple categories were scored as an average of the two categories used. The numerical value for each factor was then multiplied by an index of direct exposure to climate change based on the proportion of the species’ geographic distribution exposed to different magnitudes of changing mean annual temperature and mean annual moisture index ([30], S1 Table). The values for each factor were then summed to produce the overall index value. The NatureServe approach assigns the final numerical score to a category of climate change vulnerability (eg. moderately vulnerable). However, we here used the underlying continuous values to allow a finer grained analysis. The final index value therefore integrates information on (i) sensitivity, as estimated from biological traits, (ii) indirect exposure to climate change, as estimated from the spatial overlap between the species’ distribution and three indirect exposure factors (natural barriers, anthropogenic barriers and sea-level rise), and (iii) direct exposure to climate change as estimated from climate projections within the geographic distribution of the species. Species that have both high sensitivity and high exposure to rapid climate change ultimately score as the most climate change vulnerable. Sparse information often limited the number of factors that we could assess, but a minimum of 13 out of the 20 sensitivity and indirect exposure factors is required by the NatureServe index to estimate overall vulnerability ([26]; the factors we used are listed in Table 1). Sufficient information was available for 213 out of the 232 species initially selected (S2 Dataset). The NatureServe approach focuses on the intrinsic traits and physiological characteristics of a species and does not include geographic range size or anthropogenic threats to the species. This renders the index comparable among species with differing conservation status or geographic range size.

### Exposure

As a proxy for species’ direct exposure to climate change we used projections of mean annual temperature and mean annual moisture index under the IPCC A1F1 scenario, in which the world remains heavily reliant on fossil fuels [31] for the time horizon 2050 (S1 Table). Climatic layers were generated using the software package ANUCLIM (v6.1), based on a 9-second digital elevation model for Australia. Adapting the scheme given in Young et al [26] for an Australian context, we categorised projected change in annual mean temperature and change in mean annual moisture index according to the categories in S1 Table. A change of 0–1°C was scored as category 1, whereas a change greater than 2.25°C was scored as category 5. We then calculated the proportion of each species’ current distribution that would be affected by the different magnitudes of climate change.

Indirect exposure was assessed by estimating the extent to which each species’ current geographic distribution overlaps natural and anthropogenic barriers, or regions affected by sea level rise (S2 Table). As some species are expected to track suitable climatic conditions beyond their current distribution through dispersal, it is anticipated that species surrounded by natural and anthropogenic barriers will have greater difficulty tracking a changing climate [32–33]. Natural barriers were major water bodies (oceans and inland lakes), areas devoid of vegetation, and highlands across the continent. We extracted features categorised as ‘water’ or ‘bare ground’ from the United States Geological Survey Global Land Cover 2000 dataset (v.1; [34]), and combined these with the distribution of land greater than 700m above sea level, based on a global digital elevation model at 30m spatial resolution from the National Geophysical Data Center of the National Oceanic and Atmospheric Administration [35]. Anthropogenic barriers were approximated by mapping the distribution of urban, cultivated and managed land uses from the Global Land Cover 2000 dataset [34]. These features represent land converted in cities, urban areas and farmland, which will act as dispersal barriers for many threatened species. Proximity to natural and anthropogenic barriers was calculated by defining a 50km buffer [26] around each species’ current geographic distribution and overlaying this onto the distributions of the barriers to calculate the proportion of the buffer that overlaps with barriers (S2 Table). Species for which anthropogenic barriers were unlikely to represent a dispersal barrier (e.g. many birds, such as the Tasmanian wedge-tailed eagle *Aquila audax fleayi*) were scored as neutral (N) for this factor.

### Sensitivity

The extent to which intrinsic traits and environmental requirements render species sensitive to climate change was determined by collecting information on ten sensitivity factors (Table 1). Species were scored for each factor using information from the Australian federal government’s Species Profile and Threats Database [27], draft and approved species recovery plans, conservation and listing advice, state level species information profiles, and relevant scientific literature (S2 Dataset). Recourse to the scientific literature was necessary primarily to derive scores for dispersal ability, reliance on pollinators and genetic variability (e.g. direct genetic variation estimates, or signs of a recent genetic bottleneck) which government databases and recovery plans often lacked.

### Index variability according to range size and taxonomic group

We used an analysis of covariance (ANCOVA) to test for a possible relationship between taxonomic group and vulnerability index score, while accounting for the difference in geographic range size (log_10_-transformed). Neither geographic range size nor any other variables relating to conservation status (ie. extinction risk) are included in the vulnerability index calculation, which is founded only upon intrinsic biological variables and exposure measures [26]. This allows the analysis to assess climate change vulnerability separately from extinction risk, which also includes other threats, such as habitat loss and population declines. A one-way analysis of variance (ANOVA) was used to test for a difference in mean climate change vulnerability among taxonomic groups. All statistical tests were performed using the statistical package R version 2.13.0 [36].

### The spatial distribution of climate change vulnerability

We mapped separately the distribution of the species that are affected by each factor driving climate change vulnerability. A species was considered to be affected by a particular sensitivity factor if it was scored as ‘somewhat increased vulnerability’ (SIV) or higher for that factor, indicating that this factor is contributing to the species overall vulnerability. For indirect exposure factors, the species was only considered affected if it was scored as ‘increased vulnerability’ (IV) or higher. The rationale behind this is that indirect exposure factors (eg. sea level rise or natural barriers) generally affect a species only in some parts of its range and are not range-wide. Only including those species that were scored as at least IV, ensured that a large portion of their range was affected by an indirect exposure factor.

The distribution of species affected by the vulnerability factors were mapped using the bioregions of the Interim Biogeographic Regionalisation of Australia (IBRA, v6.1), the landscape divisions used for national conservation planning in Australia [37]. A factor was considered to be present in a given bioregion if 10% or more of the range of a species affected by that factor fell within it. Each factor was mapped separately showing the percentage of species affected by it in each bioregion, thus accounting for the different number of species present in the different bioregions. Because a species is only scored for either ‘genetic variation’ or ‘signs of recent bottlenecks’ (see Table 1) these were merged for spatial analysis under ‘low genetic variation’. The major factor driving vulnerability for any given bioregion was the one that affected the largest percentage of species.

## Results

Climate change vulnerability index values for the 213 threatened species assessed ranged from 11.3 (extremely vulnerable) for the mountain pygmy possum (*Burramys parvus*) to -5 (low vulnerability) for the western quoll (*Dasyurus geoffroii;* S3 Table). By convention, a score above four indicates moderate or high climate change vulnerability, while a value below -2.0 indicates that the species might benefit from climate change [26]. Mean vulnerability index across species was 3.6, and the median was 3.8, with most species showing intermediate levels of vulnerability and relatively few showing particularly low or high vulnerability (S3 Table). Indeed, the frequency distribution of index values was not significantly different from normal (Shapiro-Wilk test: W = 0.992, p = 0.275). Ninety-six species (45.1% of the total) had an index value exceeding 4.0, indicating that nearly half of Australia’s threatened species considered are moderately to highly vulnerable to climate change (Table 2).

**Table 2:** Number (and percentage) of threatened species within each taxon affected by each vulnerability factor.

	Plants n = 94	Amphibians n = 19	Reptiles n = 13	Birds n = 44	Mammals n = 43	All Taxa n = 213
Proximity to sea level rise	0 (0%)	0 (0%)	1 (7.7%)	2 (4.6%)	7 (16.3%)	10 (4.7%)
Proximity to natural barriers	14 (14.9%)	10 (52.6%)	2 (15.4%)	17 (38.6%)	18 (41.9%)	61 (28.6%)
Proximity to Anthropogenic barriers	17 (18.1%)	0 (0%)	1 (7.7%)	0 (0%)	2 (4.7%)	20 (9.4%)
Poor dispersal ability	57 (60.6%)	7 (36.8%)	2 (15.4%)	0 (0%)	3 (7.0%)	69 (32.4%)
Reliance on cool temperatures	11 (11.7%)	6 (31.6%)	2 (15.4%)	0 (0%)	4 (9.3%)	23 (10.8%)
Reliance on a particular moisture regime or habitat	64 (68.1%)	19 (100%)	5 (38.5%)	19 (43.2%)	20 (46.5%)	127 (59.6%)
Reliance on a particular disturbance regime	70 (74.5%)	10 (52.6%)	4 (30.8%)	28 (63.6%)	30 (69.8%)	142 (66.7%)
Reliance on snow-cover habitats	1 (1.1%)	2 (10.5%)	0 (0%)	0 (0%)	1 (2.3%)	4 (1.9%)
Reliance on a particular abiotic feature or derivative	27 (28.7%)	9 (47.4%)	4 (30.8%)	0 (0%)	14 (32.6%)	54 (25.4%)
Reliance on another species for habitat	14 (14.9%)	0 (0%)	3 (23.1%)	6 (13.6%)	7 (16.3%)	30 (14.1%)
Reliance on a particular species for diet	-	1 (5.3%)	4 (30.8%)	3 (6.8%)	5 (11.6%)	13 (6.1%)
Reliance on a particular species for pollination	23 (24.5%)	-	-	-	-	23 (10.8%)
Reliance on a particular species for propagule dispersal	8 (8.5%)	0 (0%)	0 (0%)	0 (0%)	0 (0%)	8 (3.8%)
Reliance on a particular species for other interspecific interaction	6 (6.4%)	0 (0%)	0 (0%)	0 (0%)	0 (0%)	6 (2.8%)
Low genetic variation	50 (53.2%)	12 (63.3%)	7 (53.9%)	32 (72.7%)	22 (51.2%)	123 (57.8%)
Average number of factors affecting taxon	4.447	4.526	3.231	2.727	3.535	3.693
Proportion of species with moderate to high climate change vulnerability (>4.0)	58 (61.7%)	13 (68.4%)	7 (53.9%)	4 (9.1%)	14 (32.6%)	96 (45.07%)

The factor affecting the most species in each taxonomic group is underlined. Note that columns do not sum to the number of species in the group, because each species can be affected by more than one vulnerability factor.

**Fig 1:**
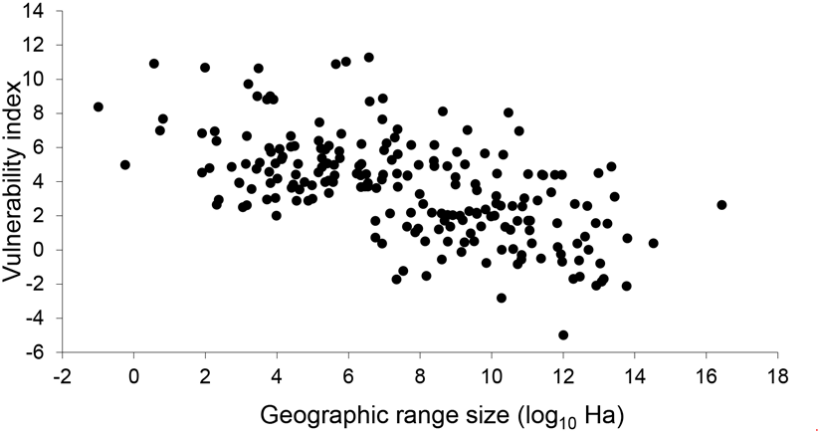
The relationship between climate change vulnerability index and geographic range size.

An ANCOVA using geographic range size (log_10_ transformed) and taxonomic group as predictors revealed that both had a significant association with the vulnerability index (F_5,204_ = 36.05, p< 0.001). Geographic range size was negatively related to climate change vulnerability, with the most narrowly distributed species showing high to extreme vulnerability (F_1,204_ = 133.64, p< 0.001; Fig 1).

**Fig 2:**
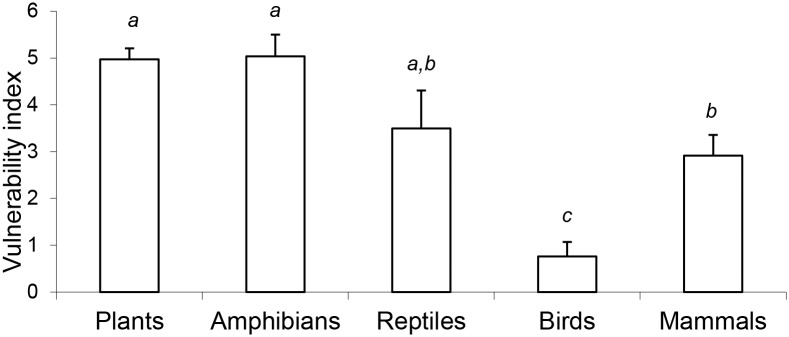
Mean climate change vulnerability for the five taxonomic groups of Australian threatened species considered in this study. Error bars represent 1 SE. Letters represent groups with no significant difference at a 95% CI, according to Tukey’s honestly significant difference test.

Climate change vulnerability varied significantly between taxonomic groups (F_4,204_ = 11.66, p< 0.001; Fig 2). Overall, amphibians were the most vulnerable group to climate change (mean = 5.0, SE = 0.5), followed by plants (mean = 5.0, SE = 0.2; Fig 2), reptiles (mean = 3.5, SE = 0.8), mammals (mean = 2.9, SE = 0.4) and finally birds (mean = 0.8, SE = 0.3).

The main factors driving climate change vulnerability were dependence on a particular disturbance regime (typically fire), reliance on a particular moisture regime or habitat, and low genetic variation (Table 2). Different factors were important for different taxonomic groups, with reliance on particular moisture regimes and low genetic variation being most important for amphibians and reptiles, reliance on disturbance regimes and low genetic variation affecting birds and mammals, and poor dispersal ability and reliance on particular disturbance and moisture regimes affecting plants (Table 2). On average, birds were affected by the smallest number of factors (2.7), whilst amphibians (4.5) and plants (4.4) were affected by the most, suggesting that these latter groups will require a more complex portfolio of management actions to help them adapt to climate change.

**Fig 3:**
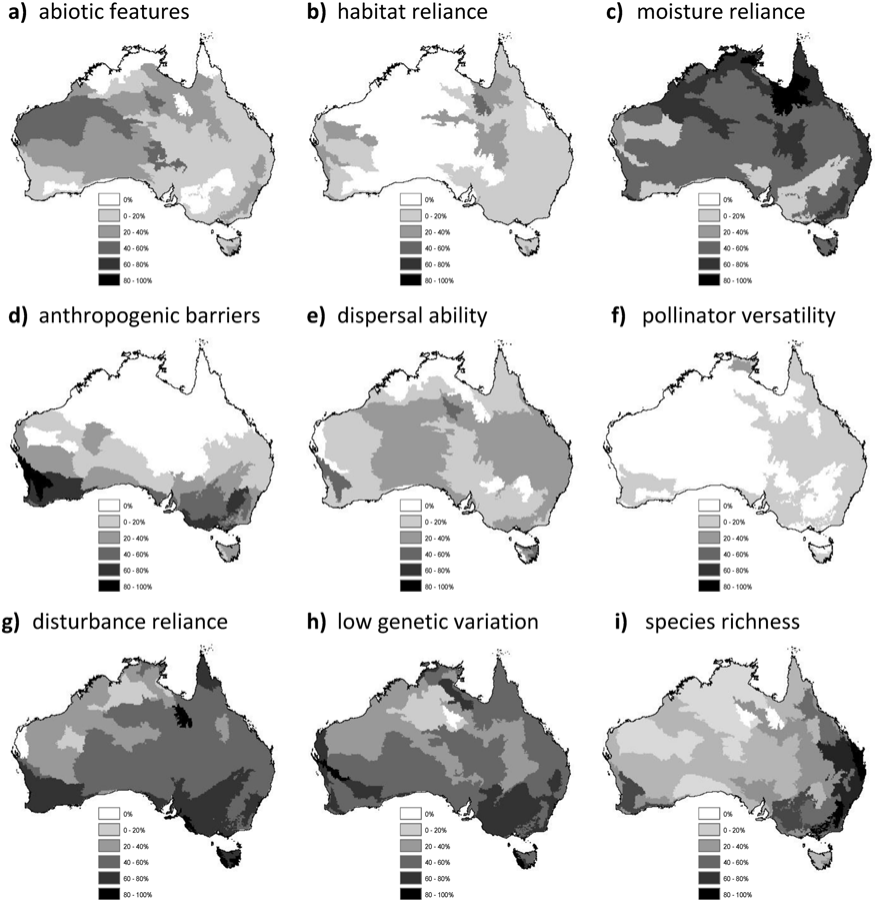
The spatial distributions of the species affected by the eight most important factors driving climate change vulnerability of threatened species in Australia. The shading darkens as the proportion of species occurring in the bioregion is affected by each factor; a) reliance on particular abiotic features or derivatives for habitat, b) reliance on other species for habitat, c) reliance on a particular moisture regime or habitat, d) proximity to anthropogenic barriers, e) poor dispersal ability, f) pollinator versatility, g) reliance on a particular disturbance regime, and h) low genetic variation. To aid in the interpretation of proportions, the distribution of threatened species richness is shown in i). The spatial distributions of species’ vulnerability to seven supplementary factors is illustrated in (S1 Fig).

Five factors stood out as having a pervasive influence on the assessed threatened species across much of Australia (Fig 3). These were reliance on a particular abiotic feature or derivative, reliance on a particular moisture regime or habitat, poor dispersal ability, reliance on a particular disturbance regime and low genetic variation (refer to Table 1 for complete descriptions of factors). Reliance on other species for propagule dispersal was uncommon among our sample of species although prevalent in the south-west corner of Western Australia and Tasmania, where two *Daviesia* species rely mainly on ants for seed dispersal [38]. Reliance on other species for pollination showed a similar pattern, affecting species along the eastern coastline, south-west Western Australia and northernmost Northern Territory. Reliance on cool temperatures, reliance on other species for diet and a reliance on species for other interactions (eg. mycorrhizal symbiosis) were all predominant factors in south-east Australia and Tasmania. A reliance of one species on other species for suitable habitat was the most prominent factor in western and north-west Queensland, while proximity to anthropogenic barriers mostly affected species along the south coast of the continent. Only the eight numerically most important factors are shown in Fig 3. Factors not shown are exposure to rising sea levels and proximity to natural barriers which affected only coastal species and those on either side of the Great Dividing Range, and reliance on snow cover which is confined to the Alps bioregion.

## Discussion

To guide effective management, climate adaptation actions must be tailored to individual or multiple vulnerability factors. It is not enough to know that a species or region is vulnerable to climate change, we must know why it is vulnerable to derive a sensible on-ground management strategy. Mapping the spatial distribution of the species affected by the various climate change vulnerability factors, as we have done here, is a crucial first step in designing effective actions. Our results revealed enormous variation in the spatial distribution of species affected by different climate change vulnerability factors, as well as substantial taxonomic heterogeneity in climate change vulnerability and its drivers.

Nearly half of the threatened species comprising our sample were vulnerable to climate change. The most vulnerable was the mountain pygmy possum, and consideration of its life history reveals a series of complex interacting factors. It is a specialist species, with low genetic variation in some populations, having undergone a significant range contraction since the last glacial maximum when it occurred throughout most of south-eastern Australia [39–40], and is now further threatened by habitat loss with the ongoing development of ski resorts, in addition to a variety of other threats. The only Australian mammal confined to the Australian Alps bioregion, the species is dependent on winter snow cover and cool temperatures [41]. Mountain pygmy possums are already responding to climate change by waking earlier from hibernation, which has led to food shortages through temporal uncoupling with the emergence of bogong moths (*Agrotis infusa*), a key post-winter food source [41]. Alpine species and habitats in general may be more at risk because of the limited options for adjustment of the geographic distribution in response to a changing climate [1, 14, 42]. Using bioclimatic modelling, Brereton et al. [1] predicted that the mountain pygmy possum would be driven extinct by a 1°C rise in temperature, and given the species current restricted distribution, it remains to be seen whether the species will survive the coming decades.

At the other end of the vulnerability scale was the western quoll, a generalist species in its dietary and habitat requirements. Despite a severe range contraction because of habitat clearance and predation from feral species, the western quoll has a high dispersal capacity and the greatest genetic variation of all quoll species [43–44].

Climate change vulnerability increases strongly as geographic range size declines. This could arise in part because threatened species that have already been heavily affected by habitat loss and fragmentation are now at increased climate change vulnerability through low genetic variation (due to population declines) or specific habitat requirements forcing the species into small fragments of their former range [45–47]. Another explanation is that some life history traits associated with high climate change vulnerability are also related to narrow geographic range size, for example habitat specialists that survive only in naturally uncommon landscapes or microhabitats [48]. Regardless of the mechanism, our finding that the most narrowly distributed species are also the most vulnerable to climate change suggests that urgent actions are needed to help these species in particular adapt to climate change.

In agreement with other studies [14,49], we found that amphibians were the most climate change vulnerable group, with heavy reliance on local moisture regimes and aquatic habitats that are likely to be negatively impacted by climate change. Altered interactions with chytrid fungus (*Batrachochytrium dendrobatidis*) and cane toads (*Bufo marinus*) due to rising temperatures are listed as key threats to Australian amphibians, where cane toads may further expand their range and the distribution of chytrid fungus may shift to new areas [14]. Plants were the second most vulnerable taxon out of the sample of species we assessed, often constrained in their distribution by physiological factors such as a reliance on a particular soil type, relatively poor dispersal ability and low genetic variation through small population size [14, 47, 50]. Unlike plants, many birds are excellent dispersers and often with less restrictive habitat requirements, rendering them the least vulnerable taxonomic group in this analysis.

Results revealed great spatial variation in the proportion of species affected by each of the major factors driving climate change vulnerability among our sample of threatened species. Species in each region were affected by different numbers and types of factors, and groups of factors appeared to operate in concert among species in different regions. The results from this study also suggest that the indices need to be decomposed into their constituent elements before they can usefully guide management actions. Comparing two different regions illustrates this point clearly. The predominant factors driving the climate change vulnerability of species along the south-east coastline of Australia are a reliance on particular disturbance regimes and low genetic variation, they are also exposed to sea level rise, anthropogenic barriers, and natural barriers comprising the ocean and the Great Dividing Range. In contrast, upper Northern Territory is predominantly impacted by reliance on particular disturbance regimes, low genetic variation, reliance on particular moisture regimes and reliance on other species for pollination. These regions will require a suite of actions that target different factors driving vulnerability to climate change. On a species level, management can become even more complicated, with some species having multiple factors contributing to their climate change vulnerability, highlighting the need to decompose vulnerability to explore the contributing factors. A good example of this is the Nielsen Park she-oak (*Allocasuarina portuensis*), which only exists as a tiny reintroduced population in Sydney’s eastern suburbs. As well as by its extremely small global population size of only a few dozen individuals, this species is affected by five different climate change vulnerability factors: limited dispersal ability, reliance on a particular soil type, low genetic variation, is surrounded by natural and anthropogenic barriers and is an obligate seed regenerator, i.e. dependent on fire to kill the adult tree and release new seed, therefore careful management of all these factors is required [51].

Management can also be targeted towards specific factors. For example amphibian species richness is largely concentrated along the south-east coastline of Australia, which makes reliance on particular moisture regimes or habitats a significant vulnerability factor there. Species affected by low genetic variation or recent population bottlenecks are prevalent along the coastline of south and south-east Australia, in both fragmented and undisturbed areas (Fig 3h). Many species in these regions have undergone major range contractions through habitat destruction and the introduction of invasive species, though their distributions once extended much further north [14]. For example, the South Australian glossy black-cockatoo (*Calyptorhynchus lathami halmaturinus*) once occurred in mainland South Australia, but is now restricted to Kangaroo Island owing to mainland habitat clearance [52]. Numbers dropped to as low as 158 birds in 1995, though the population had recovered to around 320 birds in 2006, which is suggestive of a recent bottleneck [52] potentially increasing its vulnerability to climate change. More generally, dependence on a particular disturbance regime that is likely to change with climate (e.g. fire), is driving climate change vulnerability throughout eastern South Australia, Victoria, western New South Wales and the south coast of Western Australia. In Australia, many species are reliant on appropriate fire regimes for reproduction and habitat. For example, the abundance of the Pilliga mouse (*Pseudomys pilligaensis*) increases fivefold in fire-induced regrowth forest (18–24 months post fire) in comparison with mature forest (>20years) and 28-fold in the intermediate growth stage [53]. Studies have found that populations peak 20–24 months post fire and following an above average rainfall year, though as with many opportunistic breeders, the population then declines rapidly [54]. However, mature forest is required for breeding habitat [53]. Fires are expected to become more frequent, intense and erratic as a result of climate change in Australia [43, 55–57]. Reliance on specific moisture regimes is a major factor in north-eastern Queensland and in upper Western Australia and Northern Territory. For example, the western partridge pigeon (*Geophaps smithii blaauwi*) depends on a reliable water source for survival during the late dry season [58], and reductions in rainfall and increasing temperatures as a result of climate change could pose a serious risk.

Once the drivers of climate change vulnerability are known for species, management actions can be derived (S4 Table). Actions associated with reducing vulnerability for small bodied species such as amphibians include the installation of microhabitat refuges and restoration and manipulation of moisture levels at breeding sites [59]. Artificially changing the habitat and local microclimate to be more suitable for amphibians may give them the best chance of surviving climate change. Often dispersal limited, some species may be best assisted by translocation and, in the case of plants, by replanting of seedlings at new climatically suitable sites [60, 61]. In some cases, it may be most cost effective to establish captive populations, as has recently been attempted for orange-bellied parrots [62]. It is also important to consider whether these actions will take place in protected areas, where because of shelter from other threats, there is a stronger chance of success [63].

Our analysis has revealed multiple drivers of climate change vulnerability for many of Australia’s threatened species and in many regions of Australia, suggesting that different actions will be needed in different areas and highlighting the need for a spatial prioritisation of conservation actions and focal areas. Though this sample provides a good reflection of many of Australia’s threatened flora and fauna, a full assessment of all threatened species would be worth pursuing. It is critical that recovery and management plans for threatened species are updated to include climate change vulnerability and its implications. Spatially linking actions to climate change vulnerability factors is the most direct way to improve the chance of species surviving climate change, because understanding the spatial distribution of each factor helps to spatially prioritise actions to benefit the largest number of species, making it more cost effective than considering only single species. For example, introducing a specific pollinator will only help conserve a single dependent species and is likely to be expensive, could cause unintended side effects, and might have low feasibility. On the other hand, an action such as restoration of a major vegetation type could provide benefits for multiple species. Formal analyses based on decision science will be necessary to choose among the many possible climate adaptation actions, and it will be important to consider the costs and benefits of particular actions, and how human adaptation to climate change drives future habitat loss through land use change. Given the accelerating rate of climate change and habitat loss in the 21^st^ century, no time should be spared in planning and implementing on-ground actions to get threatened species ready to face climate change.

## Supporting Information

S1 Fig The bioregional distribution of the species affected by seven supplementary factors contributing to climate change vulnerability of threatened species in Australia that were not included in Fig 3. The shading darkens as the proportion of species occurring in the bioregion is affected by each factor; a) exposure to sea level rise, b) proximity to natural barriers, c) reliance on cool temperatures, d) dependence on snow-cover habitats, e) dietary versatility, f) reliance on other species for propagule dispersal, and g) reliance on other species for other interspecific interactions. To aid in the interpretation of proportions, the distribution of threatened species richness is shown in h).
(TIF)

S1 Table Reclassified direct exposure categories for mean annual temperature and mean annual moisture index. (DOCX)

S2 Table Scoring categories for natural and anthropogenic barriers and exposure to sea level rise. (DOCX)

S3 Table Climate change vulnerability index for 213 of Australia’s threatened species. (DOCX)

S4 Table Factors affecting climate change vulnerability of threatened species in Australia and possible actions that could be used to reduce or manage vulnerability for that particular factor. (DOCX)

S1 Dataset Spreadsheet containing the scores for exposure and sensitivity factors for the 213 species assessed. (XLSX)

S2 Dataset Spreadsheet containing collated information used to score climate change vulnerability for the 213 species assessed. (XLSX)
