# Supplementary figures and images for: Mapping the Drivers of Climate Change Vulnerability for Australia’s Threatened Species
Source: PLoS One. 2015 May 27;10(5):e0124766. doi: 10.1371/journal.pone.0124766 (PMC4446039; doi:10.1371/journal.pone.0124766)

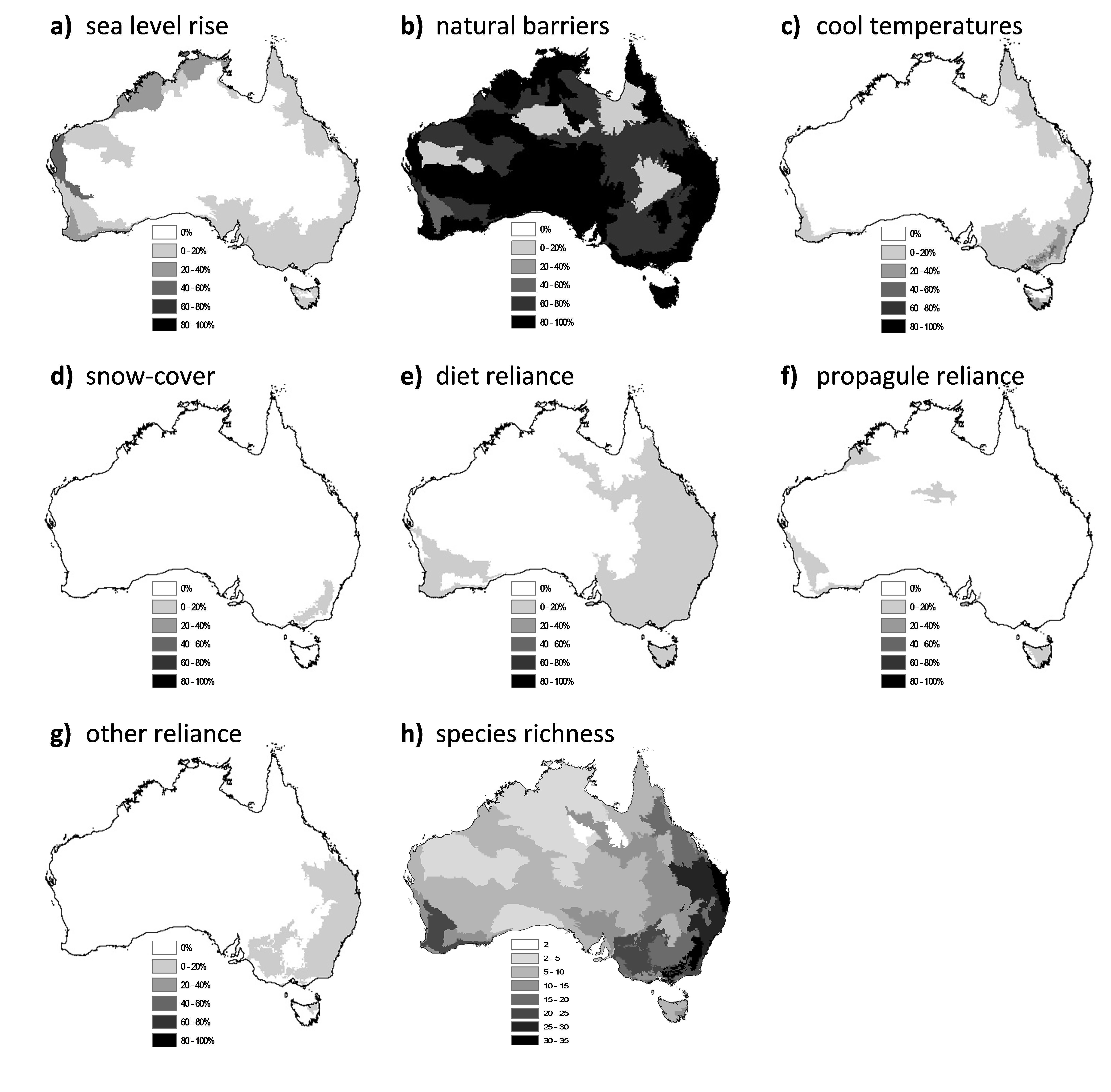

Supplement: S1 Fig — The shading darkens as the proportion of species occurring in the bioregion is affected by each factor; a) exposure to sea level rise, b) proximity to natural barriers, c) reliance on cool temperatures, d) dependence on snow-cover habitats, e) dietary versatility, f) reliance on other species for propagule dispersal, and g) reliance on other species for other interspecific interactions. To aid in the interpretation of proportions, the distribution of threatened species richness is shown in h). (TIF) [file pone.0124766.s001.tif]
