# Supplementary material for: Mapping the Drivers of Climate Change Vulnerability for Australia’s Threatened Species
Source: PLoS One. 2015 May 27;10(5):e0124766. doi: 10.1371/journal.pone.0124766 (PMC4446039; doi:10.1371/journal.pone.0124766)
Supplement: S1 Table — (DOCX) [file pone.0124766.s002.docx]

**S1 Table** Categories of direct exposure to climate change, namely the difference (absolute value) in mean annual temperature and difference in mean annual moisture index between the present time and 2050. Projections are made under the IPCC A1F scenario. Categories were based on the categories in Young *et al* (2011), but were adjusted for Australia.

| Categories | Change in mean annual temperature | Change in mean annual moisture index |
| --- | --- | --- |
| 1 | 0 – 1.0º | 0 – 0.03 |
| 2 | 1.0 – 1.5º | 0.03 – 0.06 |
| 3 | 1.5 – 2.0º | 0.06 -0.09 |
| 4 | 2.0 – 2.25º | 0.09 – 0.12 |
| 5 | > 2.25º | 0.12 – 0.15 |
| 6 |  | 0.15 – 0.19 |
