# Supplementary material for: Mapping the Drivers of Climate Change Vulnerability for Australia’s Threatened Species
Source: PLoS One. 2015 May 27;10(5):e0124766. doi: 10.1371/journal.pone.0124766 (PMC4446039; doi:10.1371/journal.pone.0124766)
Supplement: S2 Table — (DOCX) [file pone.0124766.s003.docx]

**S2 Table** Scoring categories for the factors natural and anthropogenic barriers and sea level rise, based upon those suggested by Young *et al.* 2011.

| *Categories* | *Overlap between barrier and the species distribution enlarged by a 50 km buffer* | *Percentage of a species range occurring in an area expected to be subject to sea level rise* |
| --- | --- | --- |
| **GIV** (Greatly Increase Vulnerability) | x ≥ 90% | x ≥ 90% |
| **IV** (Increase Vulnerability) | 54% ≤ x < 90% | 50% ≤ x < 90% |
| **IV/SIV** (Increase Vulnerability/ Somewhat Increase Vulnerability) | 49% ≤ x < 54% |  |
| **SIV/IV** (Somewhat Increase Vulnerability/ Increase Vulnerability) | 46% ≤ x < 49% |  |
| **SIV** (Somewhat Increase Vulnerability) | 14 ≤ x < 46% | 10 ≤ x < 49% |
| **SIV/N** (Somewhat Increase Vulnerability/ Neutral) | 10% ≤ x < 14% |  |
| **N** (Neutral) | x <10% | x <10% |
| **SDV** (Somewhat Decrease Vulnerability) | na | *Occurs in intertidal habitat, expected to increase in size*  *with rising sea level* |
