# Supplementary material for: Mapping the Drivers of Climate Change Vulnerability for Australia’s Threatened Species
Source: PLoS One. 2015 May 27;10(5):e0124766. doi: 10.1371/journal.pone.0124766 (PMC4446039; doi:10.1371/journal.pone.0124766)
Supplement: S3 Table — (DOCX) [file pone.0124766.s004.docx]

**S3 Table** Climate change vulnerability index for 213 of Australia’s threatened species, calculated according to NatureServe Guidelines (Young *et al.* 2011). Species are listed from most to least vulnerable.

| **Scientific Name** | **English Name** | **Final Index Value** |
| --- | --- | --- |
| *Burramys parvus* | Mountain Pygmy Possum | 11.275 |
| *Myriophyllum lapidicola* | Chiddarcooping myriophyllum | 11.015 |
| *Epilobium brunnescens beaugleholei* | Bog Willow-herb | 10.903 |
| *Ranunculus anemoneus* | Anemone Buttercup | 10.880 |
| *Euphrasia bowdeniae* | Euphrasia bowdeniae | 10.667 |
| *Grevillea caleyi* | Caley's Grevillea | 10.633 |
| *Borya mirabilis* | Grampians Pincushion-lily | 9.703 |
| *Daviesia cunderdin* | Cunderdin Daviesia | 9.000 |
| *Acacia pharangites* | Wongan Gully Wattle | 9.000 |
| *Pseudophryne pengilleyi* | Northern Corroboree Frog | 8.857 |
| *Lagorchestes hirsutus bernieri* | Rufous Hare-wallaby (Bernier Island) | 8.810 |
| *Lagorchestes hirsutus dorreae* | Rufous Hare-wallaby (Dorre Island) | 8.810 |
| *Pseudophryne corroboree* | Southern Corroboree Frog | 8.686 |
| *Allocasuarina portuensis* | Nielsen Park She-oak | 8.367 |
| *Litoria olongburensis* | Wallum Sedge Frog | 8.100 |
| *Carex tasmanica* | Curly Sedge | 8.032 |
| *Pseudemydura umbrina* | Western Swamp Tortoise | 7.667 |
| *Eulamprus leuraensis* | Blue Mountains water skink | 7.633 |
| *Macrozamia occidua* | Macrozamia occidua | 7.460 |
| *Tetratheca juncea* | Black-eyed Susan | 7.052 |
| *Brachyscome muelleroides* | Mueller Daisy | 7.012 |
| *Gingidia montana* | Mountain Angelica | 6.987 |
| *Mixophyes balbus* | Stuttering Frog | 6.947 |
| *Logania insularis* | Logania insularis | 6.933 |
| *Kelleria laxa* | Kelleria | 6.825 |
| *Hemiandra rutilans* | Sargents Snakebush | 6.800 |
| *Wurmbea tubulosa* | Long-flowered Nancy | 6.667 |
| *Grevillea christineae* | Christine's Grevillea | 6.667 |
| *Persicaria elatior* | Knotweed | 6.575 |
| *Ballantinia antipoda* | Southern Shepherd's Purse | 6.390 |
| *Euphrasia semipicta* | Peninsula Eyebright | 6.367 |
| *Tasmannia glaucifolia* | Fragrant Pepperbush | 6.233 |
| *Eriocaulon carsonii* | Salt Pipewort | 6.190 |
| *Litoria verreauxii alpina* | Alpine Tree Frog | 6.140 |
| *Calyptorhynchus lathami halmaturinus* | Glossy Black-cockatoo (Kangaroo Island) | 6.133 |
| *Macropus robustus isabellinus* | Barrow Island Wallaroo (Euro) | 6.095 |
| *Centrolepis caespitosa* | Matted Centrolepis | 6.078 |
| *Grevillea infecunda* | Anglesea Grevillea | 6.037 |
| *Muehlenbeckia horrida abdita* | Remote Thorny Lignum | 5.967 |
| *Petrogale persephone* | Proserpine Rock-wallaby | 5.933 |
| *Villarsia calthifolia* | Mountain Villarsia | 5.900 |
| *Geocrinia alba* | White-bellied Frog | 5.880 |
| *Hypolepis distans* | Scrambling Ground-fern | 5.832 |
| *Eleocharis keigheryi* | Keighery's Eleocharis | 5.768 |
| *Senecio macrocarpus* | Large-fruit Fireweed | 5.725 |
| *Ctenotus angusticeps* | Airlie Island Ctenotus | 5.725 |
| *Cycas megacarpa* | Cycas megacarpa | 5.638 |
| *Petrogale lateralis lateralis* | Black-flanked Rock-wallaby | 5.605 |
| *Hoplocephalus bungaroides* | Broad-headed snake | 5.573 |
| *Philoria frosti* | Baw Baw Frog | 5.452 |
| *Epacris apsleyensis* | Apsley Heath | 5.372 |
| *Taudactylus rheophilus* | Tinkling Frog | 5.363 |
| *Sagina diemensis* | Pearlwort | 5.303 |
| *Vombatus ursinus ursinus* | Common Wombat (Bass Strait) | 5.260 |
| *Sminthopsis aitkeni* | Kangaroo Island Dunnart | 5.200 |
| *Cyphanthera odgersii occidentalis* | Western Woolly Cyphanthera | 5.100 |
| *Baloskion longipes* | Baloskion longipes | 5.060 |
| *Ctenophorus yinnietharra* | Yinnietharra Rock Dragon | 5.052 |
| *Eriocaulon australasicum* | Southern Pipewort | 5.033 |
| *Stylidium coroniforme* | Wongan Hills Triggerplant | 5.033 |
| *Tasmannia purpurascens* | Broad-leaved Pepperbush | 5.033 |
| *Aprasia parapulchella* | Pink-tailed legless lizard | 4.995 |
| *Allocasuarina glareicola* | Allocasuarina glareicola | 4.973 |
| *Acacia forrestiana* | Forest's Wattle | 4.973 |
| *Astrotricha roddii* | Rod's Star Hair | 4.973 |
| *Psophodes nigrogularis nigrogularis* | Western Whipbird (Western Heath) | 4.907 |
| *Litoria piperata* | Peppered Tree Frog | 4.905 |
| *Xerothamnella parvifolia* | Ironstone Mulla Mulla | 4.900 |
| *Notoryctes typhlops* | Southern Marsupial Mole | 4.872 |
| *Potorous gilbertii* | Gilbert's Potoroo | 4.848 |
| *Stylidium galioides* | Yellow Mountain Triggerplant | 4.833 |
| *Phebalium daviesii* | Davies' Waxflower | 4.783 |
| *Geocrinia vitellina* | Orange-bellied Frog | 4.745 |
| *Pseudomys fieldi* | Shark Bay Mouse | 4.558 |
| *Hensmania chapmanii* | Chapman's Hensmania | 4.533 |
| *Lasiorhinus krefftii* | Northern Hairy-nosed Wombat | 4.515 |
| *Notoryctes caurinus* | Northern Marsupial Mole | 4.495 |
| *Maireana cheelii* | Chariot Wheels | 4.467 |
| *Xanthorrhoea bracteata* | Shiny Grasstree | 4.467 |
| *Pardalotus quadragintus* | Forty-spotted Pardalote | 4.467 |
| *Pomaderris cotoneaster* | Cotoneaster Pomaderris | 4.438 |
| *Heleioporus australiacus* | Giant Burrowing Frog | 4.438 |
| *Epacris grandis* | Grand Heath | 4.409 |
| *Chordifex abortivus* | Manypeaks Rush | 4.407 |
| *Cadellia pentastylis* | Ooline | 4.400 |
| *Litoria booroolongensis* | Booroolong Frog | 4.395 |
| *Thesium australe* | Austral Toadflax | 4.395 |
| *Delma impar* | Striped legless lizard | 4.352 |
| *Notelaea lloydii* | Lloyd's Olive | 4.352 |
| *Lasiopetalum joyceae* | Lasiopetalum joyceae | 4.352 |
| *Tetratheca glandulosa* | Glandular Pink-bell | 4.352 |
| *Pterostylis cheraphila* | Floodplain Rustyhood | 4.333 |
| *Litoria spenceri* | Spotted Tree Frog | 4.252 |
| *Atrichornis clamosus* | Noisy Scrub-bird | 4.167 |
| *Pleurophascum occidentale* | Western Giant-leaved Moss | 4.107 |
| *Pseudantechinus mimulus* | Carpentarian Antechinus | 4.058 |
| *Alectryon ramiflorus* | Isis Tamarind | 3.967 |
| *Lasiopetalum rotundifolium* | Round-leaf Lasiopetalum | 3.967 |
| *Eremophila nivea* | Silky Eremophila | 3.967 |
| *Ipomoea sp. Stirling (P.K.Latz 10408)* | Ipomoea polpha subsp. Latzii | 3.938 |
| *Daviesia pseudaphylla* | Stirling Range Daviesia | 3.907 |
| *Cycas ophiolitica* | Cycas ophiolitica | 3.900 |
| *Callistemon kenmorrisonii* | Betka Bottlebrush | 3.900 |
| *Pseudocheirus occidentalis* | Western Ringtail Possum | 3.848 |
| *Correa calycina* | Correa calycina | 3.818 |
| *Clematis fawcettii* | Stream Clematis | 3.817 |
| *Pomaderris sericea* | Bent Pomaderris | 3.773 |
| *Hydrocharis dubia* | Frogbit | 3.700 |
| *Eucalyptus cadens* | Warby Range Swamp Gum | 3.700 |
| *Tylophora linearis* | Tylophora linearis | 3.685 |
| *Haloragis exalata exalata* | Wingless Raspwort | 3.667 |
| *Leporillus conditor* | Greater Stick-nest Rat | 3.623 |
| *Spicospina flammocaerulea* | Sunset Frog | 3.610 |
| *Litoria lorica* | Armoured Mistfrog | 3.563 |
| *Perameles bougainville bougainville* | Western Barred Bandicoot (Shark Bay) | 3.535 |
| *Dasyurus maculatus gracilis* | Spotted-tailed Quoll (North QLD Subspecies) | 3.482 |
| *Pseudomys fumeus* | Smoky Mouse | 3.367 |
| *Malurus leucopterus edouardi* | White-winged Fairy-wren (Barrow Island) | 3.320 |
| *Taudactylus eungellensis* | Eungella Day Frog | 3.267 |
| *Cynanchum elegans* | White-flowered Wax Plant | 3.152 |
| *Lathamus discolor* | Swift Parrot | 3.108 |
| *Callitris oblonga* | Pygmy Cypress-pine | 3.027 |
| *Amytornis barbatus* | Grey Grasswren (Bulloo) | 3.013 |
| *Centrolepis pedderensis* | Pedder Centrolepis | 2.982 |
| *Laxmannia jamesii* | Jame's Paperlilly | 2.940 |
| *Orthrosanthus muelleri* | South Stirling Morning Iris | 2.930 |
| *Geophaps smithii smithii* | Partridge Pigeon (Eastern) | 2.882 |
| *Livistona lanuginosa* | Waxy Cabbage Palm | 2.875 |
| *Zyzomys palatalis* | Carpentarian Rock-rat | 2.875 |
| *Petrogale xanthopus xanthopus* | Yellow-footed Rock-wallaby (SA & NSW) | 2.717 |
| *Ptilotus beckerianus* | Xerothamnella parvifolia | 2.683 |
| *Sminthopsis douglasi* | Julia Creek Dunnart | 2.683 |
| *Lichenostomus melanops cassidix* | Helmeted Honeyeater | 2.633 |
| *Apium prostratum phillipii* | Fine Leaved Apium | 2.618 |
| *Callitris oblonga oblonga* | South Esk Pine | 2.618 |
| *Pandanus spiralis var. flammeus* | Edgar Range Pandanus | 2.605 |
| *Pseudomys oralis* | Hastings River Mouse | 2.573 |
| *Litoria raniformis* | Growling Grass Frog | 2.567 |
| *Denisonia maculata* | Ornamental Snake | 2.567 |
| *Litoria aurea* | Green and Golden Bell Frog | 2.538 |
| *Pimelea curviflora var. curviflora* | Pimelea curviflora var. curviflora | 2.487 |
| *Perameles gunnii gunnii* | Eastern Barred Bandicoot (Tasmania) | 2.367 |
| *Cossinia australiana* | Cossinia | 2.267 |
| *Conostylis lepidospermoides* | Sedge Conostylis | 2.167 |
| *Eremophila denticulata denticulata* | Fitzgerald Eremophila | 2.167 |
| *Calyptorhynchus banksii graptogyne* | Red-tailed Black-cockatoo (South-eastern) | 2.167 |
| *Quassia sp. Mooney Creek (J.King s.n. 1949)* | Samadera sp. Moonee Creek | 2.133 |
| *Parsonsia dorrigoensis* | Milky Silkpod | 2.133 |
| *Quassia bidwillii* | Samadera bidwillii | 2.133 |
| *Colobanthus curtisiae* | Curtis' Colobanth | 2.100 |
| *Isoodon auratus auratus* | Golden Bandicoot (mainland) | 2.035 |
| *Tyto novaehollandiae melvillensis* | Masked Owl (Tiwi Islands) | 2.023 |
| *Potorous longipes* | Long-footed Potoroo | 2.000 |
| *Neophema chrysogaster* | Orange-bellied Parrot | 2.000 |
| *Perameles gunnii unnamed subsp.* | Eastern Barred Bandicoot (Mainland) | 1.995 |
| *Litoria castanea* | Yellow-spotted Tree frog | 1.952 |
| *Pseudomys shortridgei* | Heath Rat/Mouse | 1.733 |
| *Bettongia tropica* | Northern Bettong | 1.700 |
| *Psophodes nigrogularis oberon* | Western Whipbird (Western Mallee) | 1.700 |
| *Egernia stokesii badia* | Western spiny-tailed skink | 1.700 |
| *Arenga australasica* | Australian Arenga Palm | 1.693 |
| *Sauropus macranthus* | Sauropus macranthus | 1.693 |
| *Turnix melanogaster* | Black-breasted Button-quail | 1.567 |
| *Neochmia ruficauda ruficauda* | Star Finch (Eastern) | 1.567 |
| *Leipoa ocellata* | Malleefowl | 1.533 |
| *Parantechinus apicalis* | Dibbler | 1.367 |
| *Petaurus australis unnamed subsp.* | Yellow-bellied Glider (Wet Tropics) | 1.358 |
| *Psephotus chrysopterygius* | Golden-shouldered Parrot | 1.358 |
| *Solanum karsense* | Menindee Nightshade | 1.340 |
| *Sarcochilus hartmannii* | Waxy Sarcochilus | 1.243 |
| *Pseudomys pilligaensis* | Pilliga Mouse | 1.200 |
| *Geophaps smithii blaauwi* | Partridge Pigeon (Western) | 1.170 |
| *Isoodon obesulus obesulus* | Southern Brown Bandicoot (Eastern) | 1.133 |
| *Turnix olivii* | Buff-breasted Button-quail | 1.023 |
| *Gymnobelideus leadbeateri* | Leadbeaters Possum | 1.023 |
| *Pezoporus wallicus flaviventris* | Western Ground Parrot | 0.967 |
| *Rhinonicteris aurantius (Pilbara form)* | Pilbara Leaf-nosed Bat | 0.776 |
| *Lagorchestes hirsutus unnamed subsp.* | Mala, Rufous Hare-Wallaby (central mainland form) | 0.722 |
| *Erythrotriorchis radiatus* | Red Goshawk | 0.670 |
| *Xeromys myoides* | False Water Rat | 0.500 |
| *Emydura signata* | Bellinger River Emydura | 0.500 |
| *Dasyornis longirostris* | Western Bristlebird | 0.488 |
| *Cereopsis novaehollandiae grisea* | Cape Barren Goose (South-western) | 0.433 |
| *Aquila audax fleayi* | Wedge-tailed Eagle (Tasmanian) | 0.378 |
| *Dasycercus cristicauda* | Mulgara | 0.377 |
| *Calyptorhynchus latirostris* | Carnaby's Black-cockatoo | 0.367 |
| *Delma labialis* | Striped-tailed delma | 0.367 |
| *Poephila cincta cincta* | Black-throated Finch (Southern) | 0.158 |
| *Cyclopsitta diophthalma coxeni* | Coxen's Fig-parrot | 0.043 |
| *Manorina melanotis* | Black-eared Miner | 0.000 |
| *Egernia rugosa* | Yakka skink | 0.000 |
| *Neochmia phaeton evangelinae* | Crimson Finch (White-bellied) | -0.125 |
| *Pseudomys australis* | Plains Rat | -0.258 |
| *Pezoporus occidentalis* | Night Parrot | -0.305 |
| *Falcunculus frontatus whitei* | Crested Shrike-tit (Northern) | -0.512 |
| *Myrmecobius fasciatus* | Numbat | -0.567 |
| *Stipiturus mallee* | Mallee Emu-wren | -0.567 |
| *Pteropus poliocephalus* | Grey-headed Flying-fox | -0.622 |
| *Paradelma orientalis* | Brigalow Scaly-foot | -0.700 |
| *Casuarius casuarius johnsonii* | Southern Cassowary | -0.767 |
| *Amytornis textilis modestus* | Thick-billed Grasswren (Eastern) | -0.800 |
| *Calyptorhynchus baudinii* | Baudin's Black-cockatoo | -0.843 |
| *Dasyornis brachypterus* | Eastern Bristlebird | -1.243 |
| *Onychogalea fraenata* | Bridled Nail-tail Wallaby | -1.535 |
| *Malurus coronatus coronatus* | Purple-crowned Fairy-wren (Western) | -1.575 |
| *Geophaps scripta scripta* | Squatter Pigeon (Southern) | -1.700 |
| *Pedionomus torquatus* | Plains-wanderer | -1.700 |
| *Cacatua pastinator pastinator* | Muir's Corella (Southern) | -1.733 |
| *Xanthomyza phrygia* | Regent Honeyeater | -1.865 |
| *Polytelis swainsonii* | Superb Parrot | -2.100 |
| *Acanthiza iredalei iredalei* | Slender-billed Thornbill (Western) | -2.135 |
| *Pachycephala rufogularis* | Red-lored Whistler | -2.833 |
| *Dasyurus geoffroii* | Western Quoll | -5.000 |
