# Supplementary material for: Mapping the Drivers of Climate Change Vulnerability for Australia’s Threatened Species
Source: PLoS One. 2015 May 27;10(5):e0124766. doi: 10.1371/journal.pone.0124766 (PMC4446039; doi:10.1371/journal.pone.0124766)
Supplement: S4 Table — (DOCX) [file pone.0124766.s005.docx]

**S4 Table** List of factors affecting climate vulnerability of threatened species in Australia and possible actions that could be used to reduce or manage species vulnerability for that particular factor.

| **Factor** | **Possible Actions** |
| --- | --- |
| *Sea level rise* | - Protect and restore corridors for retreat upslope or where rate of change too great, translocation |
| *Natural barriers* | - Protect and restore corridors for range shifts or where rate of change too great, translocation |
| *Anthropogenic barriers* | - Protect and restore corridors for range shifts or where rate of change too great, translocation  - Restore farm land |
| *Dispersal ability* | - Protect and restore corridors for range shifts or where rate of change too great, translocation |
| *Dependence on cool temperatures* | - Protect and restore corridors for retreat upslope to cooler habitats or where rate of change too great, translocation  - Artificial shading / increase canopy cover (Mitchell *et al.* 2008)  - Create microhabitats (e.g. rock bodies; Shoo *et al.* 2011)  - Supplement habitat (logs, boards, PVC pipes; Shoo *et al.* 2011) |
| *Dependence on moisture* | - Protect and restore moist environments particularly, streamside forests and wetlands  - Change land use and vegetation retention and restoration in catchments to reduce runoff and increase rainfall retention in soils and vegetation  - Artificial water bodies  - Portable irrigation frames or pumps (Mitchell 2001)  - Artificial misting/ sprinklers  - Employ water storage devices |
| *Dependence on disturbance regime* | - Control excessive wildfire (eg. controlled burns, decrease leaf litter)  - Artificial moisture supplementation (refer above) |
| *Dependence on snow cover* | **-** Translocation to mountains with continuing snow cover  - Create artificial snow |
| *Restriction to geological features/ derivative* | - Replicate habitat elsewhere (eg. boulder fields)  - Restoration and translocation to suitable sites |
| *Reliance on other species for habitat* | - Protect and restore corridors for range shifts or where rate of change too great, translocation of both species  - Restore degraded habitats/ breeding sites  - Introduce the relied upon species  - Artificial nests and burrows |
| *Dietary versatility* | - Introduce food sources to new areas  - Supplement diet or find suitable replacement  - Captively breed required food source and then release |
| *Pollinator versatility* | - Protect and restore corridors for range shifts or where rate of change too great, translocation of pollinators to suitable area  - Captively breed required pollinator and then release  - Find replacement pollinators |
| *Reliance on other species for propagule dispersal* | - Protect and restore corridors for range shifts or where rate of change too great, translocation of disperser species along with target species  - Translocate seeds to suitable areas |
| *Reliance on other interspecific interaction (eg. mycorrizzal symbiosis)* | - Introduce required species (eg. fungi) to habitat or new suitable area |
| *Low genetic diversity* | - Increase population size (reduce threats, captively breed)  - Increase meta-population connectivity by protecting and restoring corridors or where this is insufficient translocate/ swap individuals between populations  - Increase patch size: increase size of protected areas, restore habitats and protect refugia |

**References;**

Mitchell NJ (2001) Males call more from wetter nests: effects of substrate water potential on

reproductive behaviours of terrestrial toadlets. Proc R Soc B 268: 87-93.

Mitchell NJ, Kearney MR, Nelson NJ, Porter WP (2008) Predicting the fate of a living fossil: how will global warming affect sex determination and hatching phenology in tuatara? Proc R Soc B 275: 2185-2193.

Shoo LP, Olson DH, McMenamin SK, Murray KA, Van Sluys M, et al. (2011) Engineering a future for amphibians under climate change. J Appl Ecol 48: 487-492.
